# DYING WITH DEMENTIA: CAREGIVERS’ OBSERVATIONS OF PHYSICAL AND/OR BEHAVIORAL/PSYCHOLOGICAL DECLINE IN OLDER ADULTS

**DOI:** 10.1093/geroni/igae098.2354

**Published:** 2024-12-31

**Authors:** Cynthia Hovland, Christopher Mallett

**Affiliations:** Cleveland State University, Cleveland, Ohio, United States; Cleveland State University, Cleveland, Ohio, United States

## Abstract

Family caregivers of older persons with dementia have significant challenges across many domains. These reported problems encountered over their caregiving time are for many reasons, but what makes the caretaking difficult is complicated by both the unknown nature of the dementia disease and the dying trajectory. While there are studies, primarily from health-care professionals, of this dying process and the last few weeks of life for older persons with dementia, much less is known directly from the family caregivers’ perspectives and experiences. This qualitative study of 30 caregivers of family members aged 65 years and older who died with dementia related diagnoses used in-depth qualitative interviews conducted over a 12-month period and directed content analysis to understand the data. The study asked what physical, behavioral, and psychological changes they observed and experienced during their family members’ last weeks of life. Three primary themes were identified around behavioral and psychological changes: (a) they become different people, (b) did not recognize caregiver, and (c) wandering and getting lost; and two primary themes identified physical decline: (a) system started shutting down and (b) drastically diminished self-sufficiency. Implications for families and professionals are reviewed and discussed.

